# Hospital Construction

**Published:** 1897-12-18

**Authors:** 


### HOSPITAL CONSTRUCTION.

### THE PARK HOSPITAL.

"Mr. Edwin T. Hall, F.R.I.B.A.," architect of the Park Hospital, writes: I have read your article in your issue of 27th as to the above, and, while thanking you for your appreciation of my work, I should like to say a word as to three of your criticisms. First, as to the covered ways connecting the various buildings. This is a matter so closely affecting the comfort of the staff—in which I take the greatest interest—that I venture, with every respect, to differ from your conclusions. It is admitted that there must be a thorough aerial separation between buildings in an infectious hospital, so that totally enclosed corridors are not to be thought of—in that we all agree. But I do not agree with your view that in a covered way 10 ft. wide umbrellas and waterproofs are required, as if the staff were in the open. Why, sir, this covered way is a fixed "umbrella" 12 ft. wide, and anyone walking near the leeward edge would be effectually protected from almost anything. To further emphasise my position, permit me to quote the following from my recent address at the Leeds Congress:—

"Much has been said for and against covered ways connecting all wards with the administrative buildings. I am decidedly in favour of them, certainly in a large hospital. In such an institution it is more economical to have a centre or two from which can be supplied (1) steam, by which, in preference to coal, all the hot water required for heating the wards and for services to sinks and baths should be heated; (2) electricity for lighting, for telephones, and fire alarms; (3) water for cisterns supplying sinks and baths, fire mains, &c. The various mains and cables to convey these fluids should be carried in subways (well lighted and ventilated), by means of which they are protected from weather, and are accessible at all parts for repair. The roof of these subways forms at once the platform for a covered way, and the roofs of the covered ways, raised near the pavilions, are invaluable as balconies for the first-floor wards. On to these balconies patients' beds can be wheeled for sun baths, the curative value of which is admitted by all doctors. Covered ways should be constructed of as durable materials as the other buildings. The cost of maintenance is thereby reduced to a minimum. It is said that hospitals of detached buildings having no covered ways between them are approved by the staff, but it must be better for all to have a dry and solid pathway to walk on than a soft footway covered with snow and sludge in winter or deluged by the rain storms of the summer. The covered ways should be 8 ft. to 10 ft. wide, and at least 8 ft. high, open at the sides, but protected by a balustrade, say, 4 ft. high, kept 6 in. from the floor. It is said that the open side does not keep out the weather. True, neither does an umbrella, but except on very rare occasions both afford great protection. Ordinary rain from thunderstorms comes down, if not vertically at a small angle from the vertical, and a cover 8 ft. or 10 ft. wide would be an efficient protection from eleven-twelfths of our storms of rain, snow, or sleet. Doctors, nurses, servants, food, fuel, and all stores constantly pass from one part to another of a hospital, and need all the protection that can be given to them, and when to this is added the value of subways and sun-balconies, I venture to think the case is complete."

Next as to the cost of the hospital, which you say is "great." If that is used in a relative sense I can only say that, compared with its two or three contemporaries erected on a similar scale, the Park will be found to be the cheapest by a large percentage. The last point is as to the cost of the central stacks of glazed faïence in the large wards which you commend. It would occupy too much of your space to set out the actual calculations, but you can accept my assurance that if these stacks had been constructed of ordinary stock bricks, externally cemented and painted, the first cost would have been several pounds in excess of the cost of the faïence stack, while the area of the brick stack would have been nearly three times that of the other. The faïence is a permanent decoration; it is kept clean and bright with a sponge and water, while the cemented stack would have to be periodically repainted at considerable cost. When I designed the faïence stack the late Sir Henry Doulton took the keenest interest in it. It was as treated by me an entire novelty in fever hospitals, and he looked for its extensive adoption. Among the hundreds of experts who visited the Park Hospital during its construction there was no feature which received such careful examination as this stack of mine. The stack contains four smoke flues all swept from the basement, and eight ventilating flues, and its greatest area is 3 ft. 6 in. square.

*** We gladly publish the above letter, because it raises points of great importance in regard to hospital construction, points also which Mr. Hall has evidently very carefully considered. The question of the corridors is one that has been much discussed, and that has given rise to much difference of opinion. Some hospitals have been built with absolutely distinct pavilions, even the heating being separately and independently arranged for. In others, arranged after the pavilion type, much of the advantage of this plan has been minimised by the construction of connecting corridors which were practically closed passages. Of recent years most of the new isolation hospitals have been arranged with open-sided corridors, and we may say at once that those at the Park Hospital are very favourable examples of corridors of this type. The advantages of the solid flat roof and the substantial construction, as there seen, are very great, and if open-sided corridors are to continue, as surely is the case, we can well imagine that Mr. Hall's plans will be largely copied. Hence the importance of the subject extends far beyond the Park Hospital. We do not think, however, that the last word has yet been said on the subject of corridors. Admitting, nay, urging as we do, that a complete aërial disconnection should be made between the various pavilions, and that the object of the corridor is to effect this disconnection with the smallest possible discomfort to those who have to pass from one part of the hospital to another, we cannot but feel that it ought to be possible, by a free use of fixed and widely open louvres, to ensure a much greater protection from the weather than is obtained at the Park Hospital, together with complete aërial disconnection. It is along the walls and ceilings that air is most apt to creep, and if we remember rightly Mr. Hall has completely disconnected the nurses' home by making the corridor cease some little distance away from the door. We think it very likely that this will turn out to be the direction in which a true solution of the problem will be arrived at, and that the corridors of the future will consist of shelters with louvred sides entirely separated from the buildings at each end by a short but complete break. The Park corridors are good, but certainly not perfect. Now in regard to Mr. Hall's faïence ventilating chimneys; we have not the slightest doubt that they are extremely good, but we also have not the slightest doubt that, as we said, they "must have cost a good deal of money," and the force of this criticism, so far as it has any force, does not seem to us to be in any way lessened by the statement that the same sort of chimney built in stock bricks would have cost even more. The question is, whether the necessity of such an additional means of ventilation is so great as to make their erection worth while. Under the windows and under every bed there are inlets for fresh air, and under the windows these inlets are provided with radiators, by which the incoming air can be warmed. Besides this, every window opens both top, bottom, and middle, the upper sash falling inwards. So long as the windows are open, even by a little at the top, there should be plenty of ventilation, and we may fairly ask on what occasions are these air tubes by which the chimneys are surrounded to come into play? If it is intended to close all the windows and trust to these ventilators alone, then there will be something to discuss; but so long as the opening of two or three windows on the lee side of the ward will remove as much air as would escape up these ventilation tubes, we repeat that, beautifully contrived as these faïence chimneys are, it still remains a question whether they are so essential as to be worth the money spent upon them. No one who has watched hospital construction as we have done can fail to see and to admire the great improvements which have been introduced in recent years, and we thoroughly appreciate the feelings of an architect who endeavours to make his work as perfect as possible. At the same time, we cannot but feel that the extension of the hospital system largely depends on the cost of construction being kept as low as possible, and we think there still is a margin for saving in cost without sacrificing essential principles.—Ed. *T. H.*

